# ASO Author Reflections: Improving Management of Upper Limb Complications after Breast Cancer Treatments

**DOI:** 10.1245/s10434-021-10603-z

**Published:** 2021-08-24

**Authors:** Nur Amalina Che Bakri, Richard M. Kwasnicki, Kieran Dhillon, Naairah Khan, Omar Ghandour, Alexander Cairns, Ara Darzi, Daniel R. Leff

**Affiliations:** 1 Department of Surgery and Cancer, Imperial College London, London, UK; 2 Academic Surgical Unit, Imperial College Healthcare NHS Trust, St. Mary’s Hospital, Praed Street, London, UK

## Past

The impact of treatments on breast cancer survivors should be objectively reported given rising incidence and improvements in survival, meaning more women are living with the impact of treatment.^1^ Historically, without a focus on survivorship, monitoring of upper limb dysfunction (ULD) has been largely piecemeal and subjective.^2^ Validated approaches for measuring ULD based on introspective reporting are prone to bias. For example, the DASH (Disability of Shoulder, Arm and Hand) questionnaire reflects a person’s (possibly skewed) perception of their own functionality. Current objective indicators, such as arm volume (lymphedema), on the other hand, do not represent functional morbidity and provide poor comparison between patients.^3^

## Present

Technological advances have led to the development of non-invasive and non-obtrusive wearable activity monitors (WAMs) for tracking physical activity. In oncology trials, WAMs are being used to study the association between physical activity and outcomes.^4^ Increased physical activity has been linked to better cancer patient outcomes with improvement in quality of life, complication rates and hospital length of stay.^3,5^ We recently assessed the feasibility of using WAMs to objectively monitor upper limb functional recovery after different types of breast cancer treatments.^6^ This study investigated upper limb activities in breast cancer cohort. The findings demonstrated a reduction in arm function on the operated side, followed by a slow recovery that does not return to baseline even at 2 weeks after surgery. During the 2-week post-operative period, the unoperated side was more active than the operated side. Since the pattern of post-operative physical morbidity is as predicted, i.e. a post-operative drop followed by a progressive recovery to baseline, construct validity has been established. Physical activity data measured by WAMs seem to be able to differentiate between surgical procedures.^6^ The concurrent validity of WAMs was demonstrated by the moderate negative correlation between activity levels and functional (DASH) surveys. This demonstrates the utility of the WAMs as an objective tool for assessing functional morbidity.

## Future

This research will be a starting point for future research into how WAMs may help with feedback-enabled prehabilitation and rehabilitation. A screening tool to identify individuals at risk of developing upper limb complications or requiring additional support can be developed in conjunction with engineers and physiotherapists by measuring and characterizing upper limb morbidity. Patients’ upper limb activities can be tracked as part of an enhanced recovery care plan to encourage them to take charge of their own care, led by their own individualized activity goals. This is an important step towards improving outcomes and quality of life for breast cancer survivors.

## Disclosure

No conflicts of interest to declare.
